# Occupationally Acquired HIV Infection Among Health Care Workers — United States, 1985–2013

**Published:** 2015-01-09

**Authors:** M. Patricia Joyce, David Kuhar, John T. Brooks

**Affiliations:** 1 Division of HIV/AIDS Prevention, National Center for HIV/AIDS, Viral Hepatitis, STD, and TB Prevention, CDC; 2 Division of Healthcare Quality Promotion, National Center for Emerging and Zoonotic Infectious Diseases, CDC

Case investigations of human immunodeficiency virus (HIV) infection in health care workers (HCWs) possibly acquired by exposure to HIV in the workplace are conducted by state health department HIV surveillance staff members with assistance from CDC. Since 1991, reports of occupationally acquired HIV in HCWs have been recorded by the National HIV Surveillance System following a standardized case investigation protocol. HCWs are defined as all paid and unpaid persons working in health care settings with the potential for exposure to infectious materials (e.g., blood, tissue, and specific body fluids) or contaminated medical supplies, equipment, or environmental surfaces. HCWs can include but are not limited to physicians, nurses, dental personnel, laboratory personnel, students and trainees, and persons not directly involved in patient care (e.g., housekeeping, security, and volunteer personnel). In 1987, CDC recommended the use of “universal precautions,” which became a part of “standard precautions” in 1995, to prevent occupational HIV exposures. Since 1996, occupational postexposure prophylaxis with antiretrovirals to prevent infection has been recommended.

A confirmed case of occupationally acquired HIV infection requires documentation that seroconversion in the exposed HCW is temporally related to a specific exposure to a known HIV-positive source. An HCW should immediately report an exposure event to a supervisor or facility-designated person in accordance with the institution’s infection control procedures. The serostatus of the source patient and of the exposed HCW should be documented at the time of the exposure and, exposed HCWs should be counseled on risk and offered postexposure prophylaxis as appropriate.

A possible case of occupationally acquired HIV infection is defined as an infection in an HCW whose job duties might have exposed the HCW to HIV but who lacks a documented workplace exposure. If the HIV status of the source patient is unknown or the HCW’s seroconversion after exposure was not documented as temporally related, occupational acquisition of HIV infection is possible but cannot be confirmed.

During 1985–2013, 58 confirmed and 150 possible cases of occupationally acquired HIV infection among HCWs were reported to CDC; since 1999, only one confirmed case (a laboratory technician sustaining a needle puncture while working with a live HIV culture in 2008) has been reported (1; Division of HIV/AIDS Prevention, National Center for HIV/AIDS, Viral Hepatitis, STD, and TB Prevention, CDC, unpublished data, 2014) (Figure). Among the 58 confirmed cases, the routes of exposure resulting in infection were: percutaneous puncture or cut (49 cases), mucocutaneous exposure (five), both percutaneous and mucocutaneous exposure (two), and unknown (two). A total of 49 HCWs were exposed to HIV-infected blood, four to concentrated virus in a laboratory, one to visibly bloody fluid, and four to unspecified body fluids. Occupations of the HCWs with confirmed or possible HIV infection have varied widely (Table).

CDC recommends the use of standard precautions to prevent exposure of HCWs to potentially infectious body fluids when working with any patient, whether known to be infected with HIV or not (2). HCWs should assume that body fluids from all patients are infectious even if the patients are not known to be infected with HIV. Proper implementation of standard precautions (e.g., use of safety devices and barriers such as gloves and goggles) minimizes exposure risk. To prevent unintentional puncture injuries, CDC recommends a comprehensive prevention program consistent with requirements of the Occupational Safety and Health Administration’s bloodborne pathogens standard.* Medical devices engineered for sharps† protection (e.g., needleless systems) should be used. Used devices such as syringes or other sharp instruments should be disposed of in sharps containers without any attempt to recap needles. HCWs should immediately wash hands and other skin surfaces after contact with blood or body fluids. Although preventing exposures to blood and body fluids is the most important strategy for preventing occupationally acquired HIV, when occupational exposures do occur, appropriate postexposure management is critical. Guidelines for the management of occupational exposures to HIV and recommendations for postexposure prophylaxis have been published (3).

Documented occupational acquisition of HIV infection in HCWs has become rare in the United States. Few confirmed cases have been reported since the late 1990s. Whereas the paucity of cases could be the result of underreporting, it might indicate the effectiveness of more widespread and earlier treatment to reduce patient viral loads, combined with prevention strategies such as postexposure management and prophylaxis as well as improved technologies and training to reduce sharps injuries and other exposures. All cases of suspected occupationally acquired HIV infection in HCWs need to be promptly reported to state health department HIV surveillance staff and the CDC coordinator for Cases of Public Health Importance, Division of HIV/AIDS Prevention, at 404-639-0934 or 404-639-2050.

## Figures and Tables

**FIGURE:**
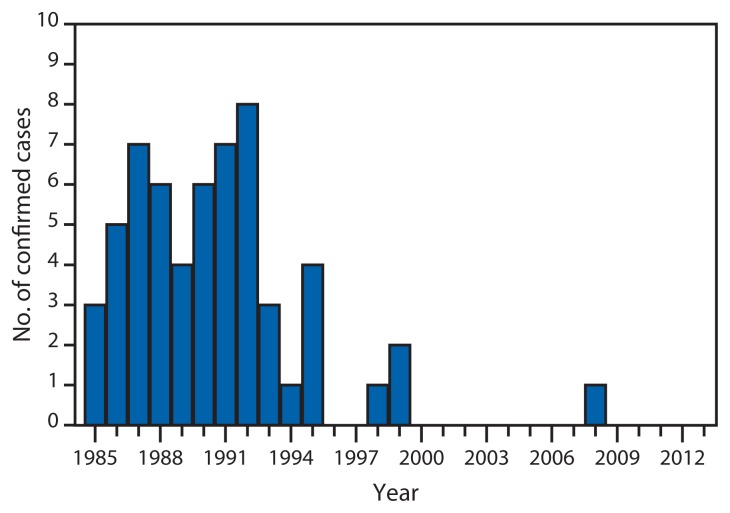
Number of confirmed cases (N = 58) of occupationally acquired HIV infection among health care workers reported to CDC — United States, 1985–2013 **Abbreviation:** HIV = human immunodeficiency virus. **Source:** Division of HIV/AIDS Prevention, National Center for HIV/AIDS, Viral Hepatitis, STD, and TB Prevention, CDC.

**TABLE:** Number of confirmed or possible cases of occupationally acquired HIV infection among health care workers reported to CDC — United States, 1985–2013

Occupation	Confirmed (N = 58)		Possible (N = 150)	
	
	No.	(%)	No.	(%)
Nurse	24	(41.4)	37	(24.7)
Laboratory technician, clinical	16	(27.6)	21	(14.0)
Physician, nonsurgical	6	(10.3)	13	(8.7)
Laboratory technician, nonclinical	4	(6.9)	—	—
Housekeeper/maintenance	2	(3.4)	14	(9.3)
Technician, surgical	2	(3.4)	2	(1.3)
Embalmer/morgue technician	1	(1.7)	2	(1.3)
Hospice caregiver/attendant	1	(1.7)	16	(10.7)
Respiratory therapist	1	(1.7)	2	(1.3)
Technician, dialysis	1	(1.7)	3	(2.0)
Dental worker, including dentist	—	—	6	(4.0)
Emergency medical technician/paramedic	—	—	13	(8.7)
Physician, surgical	—	—	6	(4.0)
Technician/Therapist, other	—	—	9	(6.0)
Other health care occupations	—	—	6	(4.0)

**Abbreviation:** HIV = human immunodeficiency virus.

**Source:** Division of HIV/AIDS Prevention, National Center for HIV/AIDS, Viral Hepatitis, STD, and TB Prevention, CDC.

## Footnotes

* 29 CFR 1910.1030.

† Needles, blades, broken glass, and other sharp objects.
